# Increased Loading Rates During Gait Correlate With Morphology of Unaffected Hip in Juveniles With Treated Developmental Hip Dysplasia

**DOI:** 10.3389/fbioe.2021.704266

**Published:** 2021-07-21

**Authors:** Wei-Chun Lee, Tsan-Yang Chen, Li-Wei Hung, Ting-Ming Wang, Chia-Hsieh Chang, Tung-Wu Lu

**Affiliations:** ^1^Department of Biomedical Engineering, National Taiwan University, Taipei, Taiwan; ^2^Department of Pediatric Orthopedics, Chang Gung Memorial Hospital, Taoyuan, Taiwan; ^3^Department of Orthopedics, Shin Kong Wu Ho-Su Memorial Hospital, Taipei, Taiwan; ^4^Department of Orthopedic Surgery, School of Medicine, National Taiwan University, Taipei, Taiwan

**Keywords:** gait, hip loading, premature osteoarthritis, hip dysplasia, hip biomechanics

## Abstract

Long-term follow-up studies on children with surgically treated developmental dysplasia of the hip (DDH) have shown that not only the affected side progresses to premature osteoarthritis, but the unaffected side may also suffer from insidious hip dysplasia or osteonecrosis. The current gait analysis study identified the loading and unloading rates of the ground reaction forces (GRF) and lower limb joint axial forces during gait, and their correlations with the hip morphology in twenty juvenile patients with surgically treated unilateral DDH during toddlerhood. In a gait laboratory, each subject walked at preferred speed on a 10-m walkway while the kinematics and the GRF were measured. Loading and unloading rates of the vertical GRF and the joint axial forces were obtained as the maximum instantaneous slope of these force curves. Radiographic measurements of the hips were taken, and the correlations between the morphological parameters and the loading and unloading rates were obtained. The patients showed greater-than-normal peak loading rates of the joint axial forces, and the loading rates on both the affected and unaffected sides were strongly correlated to the acetabular index on the unaffected side, which was also significantly correlated with the peak unloading rates on the affected side. These results suggest that apart from regular follow-up of the affected hip, routine assessment of the morphological changes and/or increased loading rates on the unaffected hip is also important for early identification of any signs of insidious hip dysplasia and risk of premature degeneration of the cartilage.

## Introduction

Developmental dysplasia of the hip (DDH) is one of the most common pediatric hip disorder needing surgical intervention (Feeley et al., 2014). Early diagnosis before 6 months of age and early treatment with a harness is the gold standard management for this disease (Barlow, 1962). In case of late diagnosis or failure to manage with a harness, closed or open reduction with or without pelvic osteotomy would be needed to correct the abnormal morphology of the hip (Danielsson, 2000; Terjesen and Horn, 2020). Generally, even if hip dislocation was treated successfully by surgery either by closed or open reduction, there are usually remaining problems such as osteonecrosis in the femur, residual acetabular dysplasia, or residual subluxation of the hip (Roposch et al., 2013). Previous long-term follow-up studies on children treated for DDH with surgical reduction showed that not only the affected side would progress to premature osteoarthritis (OA), but that the unaffected side would also suffer from insidious hip dysplasia or osteonecrosis (Song et al., 2008; Terjesen, 2014). These problems are often a result of the residual hip morphological changes, and of the magnitudes and loading rates of the forces transmitted at the joint even after surgery. However, clinical follow-up has focused mainly on morphological changes of the affected hip joint using plain-film X-ray images of the pelvis. Monitoring the changes in the morphology and loading conditions at both hips may provide more complete information for early detection of signs of increased risk of premature OA in patients treated for DDH.

Hip dysplasia has been shown to be a pre-osteoarthritic condition leading to premature radiological hip OA (Wiberg, 1939; Jacobsen and Sonne-Holm, 2004). Morphological changes in dysplastic hips, such as reduced hip coverage and abnormal orientation of the acetabulum, will alter the loading conditions, load-transferring areas, and the lines-of-action and lever arms of the muscles, etc., leading to early degeneration (Albinana et al., 2004; Thomas et al., 2014; Wang et al., 2016; Vafaeian et al., 2017). It has been shown that in adult acetabular dysplasia, each degree reduction in lateral center-edge angle (CEA), an index of hip coverage, was associated with a 13% increase in the risk of radiographic OA (Thomas et al., 2014). In a 40-years follow-up for residual acetabular dysplasia in children with surgically treated DDH (Albinana et al., 2004), the orientation of the acetabulum using acetabular index (AI) measured on early radiographs was found to be predictive for Severin grade, an index measured on adult radiographs to indicate the risk of hip OA (Ward et al., 1997). It was also reported that an AI of 35° or more at 2 years after reduction in childhood was associated with an 80% probability of becoming a Severin grade III/IV hip in adults (Albinana et al., 2004). Apart from the acetabular morphology, that of the proximal femur, such as the neck-shaft angle (NSA), is also an important contributing factor to the increased loading rates and the risk of osteonecrosis and/or hip OA in patients with surgically treated DDH (Wang et al., 2016). Therefore, after surgical treatment of children with DDH, regular radiographic follow-up for hip morphological changes is important to identify residual hip dysplasia or osteonecrosis until growth maturity.

Apart from residual hip morphological changes, loading at higher rates at the tissue level can generate surface fissuring of cartilage, which can propagate if the joint surface is subjected to repetitive loads (Ewers et al., 2002; Kerin et al., 2003). Higher loading rates not only initiate OA at the joint, but may also accelerate fibrillation of already damaged cartilage, as observed in severe OA (Kerin et al., 2003). For the joints of the lower extremities, repetitive loadings at high loading rates occur mainly at heel-strike during gait. The swift increase in the GRF around heel-strike (also called heel-strike transient) is associated with the impact of the foot when its velocity is rapidly brought to zero (Jefferson et al., 1990). By modifying the rate at which the limb and thus its end-point (foot) descends to the ground, the GRF loading rates can be controlled (Jefferson et al., 1990; Chang et al., 2012). On the other hand, compromised swing-limb control may lead to increased loading rates of the GRF, and thus of those at the lower limb joints (Chang et al., 2012). Therefore, precise end-point (foot) control of the lower limb during the swing phase is critical for a smooth heel contact around heel-strike to reduce the ground reaction force (GRF) loading rates.

Overall gait speed can also affect the magnitudes of loading rates of the lower extremities. Previous studies on healthy adults showed that walking speed had the highest correlation with loading rate (r = 0.95) while stride length had the lowest (r = 0.85) (Collins and Whittle, 1989). In adults with ignored or partially treated unilateral DDH, altered gait spatiotemporal parameters were reported, including slower-than-normal walking speed and shorter steps, apart from joint motion deviations such as reduced hip flexion/extension, but greater knee and ankle flexion (RomanÒ et al., 1996; Lai et al., 1997; Pedersen et al., 2004). In contrast, children with treated unilateral DDH were found to have gait spatiotemporal parameters, including walking speed, similar to their healthy peers (Ömeroğlu et al., 2008; Chang et al., 2011, 2012; Wang et al., 2016). However, they still displayed asymmetrical residual gait deviations with a more flexed posture at the knee and ankle in the affected limb as a result of altered pelvic motions, namely more anterior tilt, hiking at the affected side and rotation toward the unaffected side of the pelvis (Chang et al., 2012). Event at normal gait speed, an increased loading rate was noted on both the affected and unaffected side in adolescents who had been treated for unilateral DDH by Pemberton’s osteotomy during toddlerhood (Chang et al., 2011) and those with Type II osteonecrosis at the treated hip (Wang et al., 2016). Long-term monitoring of the loading rates of the GRF and joint forces in patients with DDH was thus suggested for early detection of any risk of premature OA (Chang et al., 2011).

While morphological changes and increased loading rates have separately been related to increased risk of premature OA of the hip, no study has examined whether the increased bilateral loading rates during walking might be related to the morphology of the hips in juveniles after DDH surgery. The purpose of the current study was thus to identify the loading and unloading rates of the GRF and joint axial forces in the lower limbs, as well as spatiotemporal parameters during gait, and the possible correlations of the loading and unloading rates with the bilateral hip morphology in juveniles who had been treated for DDH during toddlerhood. It was hypothesized that the patients would show spatiotemporal gait parameters similar to those of healthy controls, but greater loading and unloading rates of the GRF and joint axial forces in both of the lower limbs, and that the loading and unloading rates would be correlated with the morphology of the affected hip.

## Materials and Methods

**FIGURE 1:**
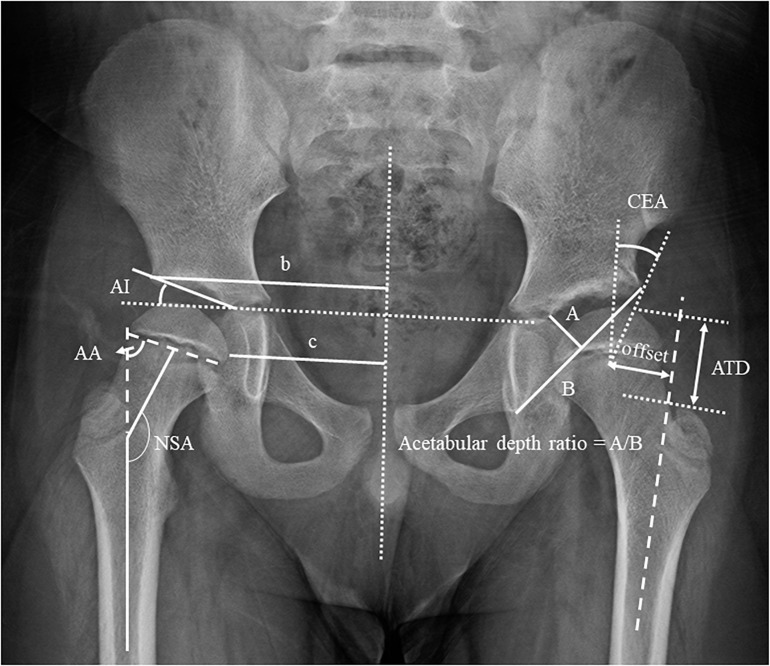
Definitions of the radiographical measurements of the morphology of the hip. NSA, neck-shaft angle; AI, acetabular index; CEA, center-edge angle; A/B, acetabular depth ratio; ATD, articulotrochanteric distance; c/b ratio; AA, Alsberg angle.

All experiments of the current study were conducted with the approval of Chang Gung Memorial Hospital Institutional Review Board (IRB No. 201601982B0C501). All the experiments and procedures conformed to the Ethical Principles for Medical Research Involving Human Subjects (World Medical Association Declaration of Helsinki). Twenty patients (DDH group; 20 females; age: 6.7 ± 2.3 years; height: 117.6 ± 16.2 cm; mass: 24.3 ± 8.1 kg; leg length discrepancy: 0.5 ± 0.3 cm) who had been treated for unilateral developmental dislocation of the hip by reduction surgery at an age of 2.3 ± 1.5 years participated in the current study (Table 1). Written informed consents were obtained from the participants and their parents or guardians as approved by the Institutional Review Board. Of the 20 subjects in the DDH group, two had been treated with closed reduction, two with open reduction, and 16 had been treated by open reduction with Pemberton’s osteotomy according to the severity. All subjects were able to walk without support, and were free of pain and infection of the hip or any other neuromuscular diseases that might influence ambulation. Follow-up radiographs showed that frontal-plane acetabular coverage was within normal range at the time of the gait experiment in all the subjects. For each subject, the NSA, AI, CEA, femoral offset, acetabular depth ratio, articulotrochanteric distance (ATD), Alsberg angle (AA), and c/b ratio (the ratio of the distance from the most medial femoral metaphysis to the pelvic midline and the distance from P-line to pelvic midline) were measured from anteroposterior X-ray images of the pelvis for both sides at the time of the gait experiment (Figure 1 and Table 1). Participants were excluded from the study if they had other neuromusculoskeletal diseases or neurological pathology that might affect gait. Thirteen healthy children (Control group; 10 girls and three boys; age: 7.8 ± 1.88 years; height: 125.8 ± 12.1 cm; mass: 26.6 ± 6.2 kg; leg length discrepancy: 0.3 ± 0.4 cm) were also recruited with the same consent procedure and served as the control group matching with the DDH group without significant between-group differences in sex, age, height, and body weight (BW). An *a priori* power analysis based on pilot results using G^∗^POWER 3 (Faul et al., 2007) determined that five subjects for each group would yield a power of 0.8 at a significance level of 0.05. Thus, the number of subjects for each group was more than adequate for the main objectives of the current study.

**TABLE 1:** Means (standard deviations) of the demographic characteristics of the Control group and the juvenile patients treated for unilateral developmental dysplasia of the hip (DDH) during toddlerhood, as well as the morphological parameters of the hips in the DDH group.

Demographic characteristics			

	DDH	Control	*p*-Value
Age at gait experiment (years)	6.7 (2.3)	7.8 (1.9)	0.119
Age at surgery (years)	2.3 (1.5)	–	–
Body mass (kg)	24.3 (8.1)	26.6 (6.2)	0.401
Body height (cm)	117.6 (16.2)	125.8 (12.1)	0.124
Body mass index	17.4 (3.5)	16.7 (2.6)	0.536
Leg length discrepancy (cm)	0.5 (0.3)	0.3 (0.4)	0.111

**Morphological parameters in DDH**			

	**Affected hip**	**Unaffected hip**	***p*-Value**

Offset (mm)	21.5 (8.2)	22.3 (5.4)	0.501
Neck-shaft angle (°)	144.5 (11.1)	143.7 (9.5)	0.781
Acetabular index (°)	18.8 (8.0)	18.7 (6.0)	0.939
Center-edge angle (°)	21.8 (11.1)	21.0 (9.4)	0.792
Acetabular depth ratio (mm/mm)	0.240 (0.047)	0.260 (0.042)	0.196
Articulotrochanteric distance (mm)	19.8 (5.2)	18.9 (5.5)	0.470
c/b ratio (mm/mm)	0.671 (0.052)	0.678 (0.050)	0.579
Alsberg angle (°)	70.9 (10.1)	68.5 (10.2)	0.379

In a hospital gait laboratory, each subject walked several times on a 10-m walkway at their preferred gait speed before data collection. To track the segmental motions of the pelvis-leg apparatus, light-weight retroreflective markers were attached to the anterior and posterior superior iliac spines, greater trochanters, mid-thighs, medial and lateral femoral epicondyles, fibular heads, tibial tuberosities, medial and lateral malleoli, navicular tuberosities, 5th metatarsal bases, big toes, and heels (Cappozzo et al., 1995). Three-dimensional trajectories of the markers were measured using a 7-camera motion analysis system (MX T-40, Vicon Motion Systems Ltd., United Kingdom) and the GRFs were measured using three forceplates (AMTI, United States). At least six successful trials, each with a complete gait cycle for each limb, were obtained for each subject for subsequent analysis.

From the measured marker and forceplate data, the angular motions, and resultant forces at the lower limb joints were calculated using inverse dynamics analysis with the pelvis-leg apparatus modeled as a 7-rigid-link system (Lu et al., 1997; Lu et al., 1998; Chang et al., 2011; 2012; Wang et al., 2016). Before the analysis, the measured GRF and marker data were low-pass filtered with a 4th order Butterworth filter with cut-off frequencies of 25 and 10 Hz, respectively. Each body link was embedded with a local coordinate system with the positive X-, Y-, and Z-axes directed anteriorly, superiorly, and to the right, respectively (Wu and Cavanagh, 1995) and a Z–X–Y Cardanic rotation sequence was used to calculate the joint angles (Grood and Suntay, 1983). The center of rotation of the hip was estimated using a functional method (Leardini et al., 1999), and those of the knee and ankle were defined as the mid-points between the lateral and medial femoral epicondyles and between the lateral and medial malleoli, respectively (RomanÒ et al., 1996). Body segmental inertial properties needed for joint force calculations were obtained using an optimization-based method (Chen et al., 2011) and the effects of soft tissue artifacts were reduced using a global optimization method (Lu and O’Connor, 1999). The axial components of the joint forces were calculated by projecting the resultant forces onto the long axis of the distal segment. Loading rates of the vertical GRF and the joint axial forces during initial contact and loading response phases, and their unloading rates during the pre-swing phase, were obtained as the maximum instantaneous slope of these force curves, calculated by finding the first derivatives of the spline curves fitted to the data using GCVSPL (Woltring, 1986; Wang et al., 2016). All the force-related variables were normalized to body weight. Loading rate variables were averaged between left and right limbs for the healthy control group. Spatiotemporal gait parameters, namely stride length, stride time, step length, step width, cadence, and walking speed were also obtained.

Each of the calculated variables between affected and unaffected sides was compared using paired *t*-tests. Comparisons between Affected vs. Control and Unaffected vs. Control were performed using independent *t*-tests. The associations between each of the radiographic measurements, and the peak loading and unloading rates of the vertical GRFs, as well as the joint axial forces for both the affected and unaffected sides were obtained using Pearson’s correlation analysis. A correlation coefficient of 0.7–0.9 indicated strong correlation, 0.4–0.6 moderate, and 0.1–0.3 weak correlation. All significance levels were set at α = 0.05. All statistical analyses were performed using SPSS 20.0 (SPSS, IBM, Armonk, New York, United States).

## Results

**FIGURE 2:**
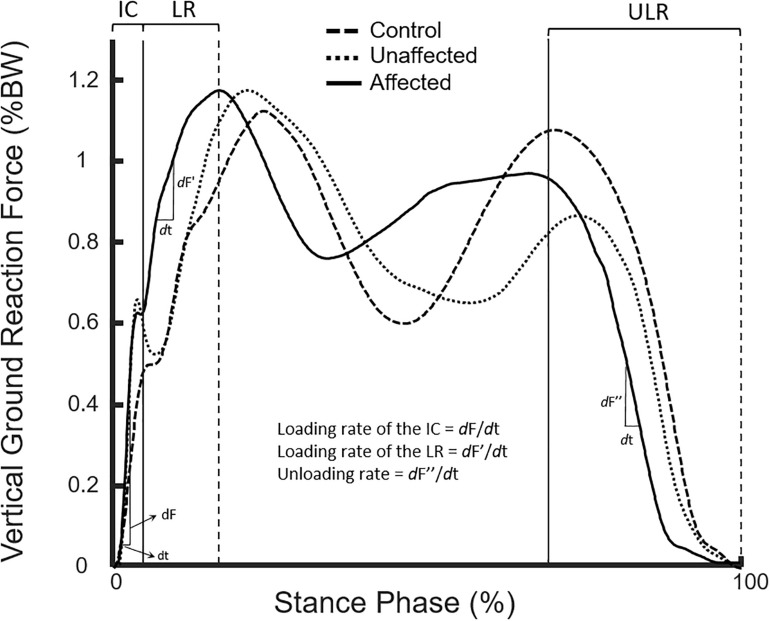
Curves of body-weight-normalized vertical ground reaction forces of the affected (solid line) and unaffected (dotted line) limbs of typical subjects in the DDH and Control group (dash line) during the stance phase of level walking.

In the patient group, no significant differences were found in the morphological parameters between affected and unaffected sides (Table 1). Leg lengths of both sides were measured from scanography, and the mean leg length discrepancies were 0.5 cm, which was not clinically significant (Table 1). The initial DLS of the affected limb was 1% longer than the unaffected limb (*p* = 0.003). No significant between-side differences were found in the gait spatiotemporal parameters. No significant differences were found in the loading rates of the joint axial forces and vertical GRF between affected and unaffected sides (Tables 2, 3 and Figure 2).

**TABLE 2:** Means (standard deviations) of gait spatiotemporal parameters for the Control group and the juvenile patients treated for unilateral developmental dysplasia of the hip (DDH) during toddlerhood.

	DDH		Control	*p-*value Affected vs. Unaffected	*p-*value (DDH vs. Control)	
				
	Affected limb	Unaffected limb			Affected vs. control	Unaffected vs. control
Walking speed (m/s)	0.63 (0.11)		0.67 (0.08)	*–*	0.216	
Cadence (step/min)	78.0 (13.5)		81.9 (8.5)	*–*	0.356	
Step width (cm)	9.3 (2.5)		9.4 (3.1)	*–*	0.925	
Stride length (m)	0.98 (0.05)	0.97 (0.07)	0.99 (0.09)	0.700	0.714	0.664
Stride time (s)	1.1 (0.2)	1.1 (0.1)	0.90 (0.1)	0.409	**<0.001**	**<0.001**
Stance (%)	60.1 (1.7)	59.9 (1.6)	60.5 (1.6)	0.431	0.159	0.927
Initial double-limb support (%)	11.4 (2.0)	10.2 (0.8)	10.7 (1.5)	**0.003**	0.178	0.111
Terminal double-limb support (%)	9.9 (0.8)	9.8 (1.5)	10.5 (1.5)	0.539	0.073	0.306

*Bold: *p* < 0.05.*

*BW, body weight.*

**TABLE 3:** Means (standard deviations) of the peak loading and unloading rates of the joint axial forces and vertical ground reaction forces (GRF).

	DDH patients		Healthy controls	*p-*value Affected vs. Unaffected	*p-*value Affected vs. Controls	*p-*value Unaffected vs. Controls
	
	Affected limb	Unaffected limb				

Loading rates (BW/s) at initial contact						
Hip force	47.2 (17.41)	45.1 (17.27)	41.4 (6.38)	0.471	**0.041**	0.140
Knee force	51.2 (18.16)	48.8 (18.32)	43.8 (7.00)	0.442	**0.027**	0.093
Ankle force	50.7 (17.85)	48.1 (18.54)	43.0 (6.52)	0.454	**0.025**	0.098
Vertical GRF	54.6 (23.88)	54.1 (19.98)	49.7 (7.82)	0.878	0.128	0.131

**Loading rates (BW/s) during loading response phase**						

Hip force	13.7 (7.81)	13.9 (4.63)	14.0 (3.23)	0.904	0.926	0.890
Knee force	14.5 (7.51)	14.4 (4.92)	13.9 (3.61)	0.861	0.705	0.960
Ankle force	12.3 (6.57)	12.5 (4.68)	12.6 (6.42)	0.927	0.895	0.942
Vertical GRF	12.5 (7.36)	14.7 (14.14)	13.0 (6.92)	0.844	0.661	0.446

**Unloading Rates (BW/s) During Pre-Swing Phase**						

Hip force	17.6 (5.35)	17.9 (6.72)	18.8 (3.34)	0.688	0.888	0.907
Knee force	16.3 (5.16)	16.0 (5.78)	16.6 (2.52)	0.534	0.802	0.836
Ankle force	14.7 (4.03)	14.4 (4.49)	14.7 (2.14)	0.663	0.789	0.701
Vertical GRF	16.0 (4.80)	15.9 (4.69)	18.2 (2.78)	0.677	0.339	0.295

*Bold: *p* < 0.05.*

*BW, body weight.*

Compared to the healthy group, the stride time of the patient group was longer than that of the healthy control group (*p* < 0.05). No significant between-group differences were found for any other spatiotemporal parameters. Compared to the healthy controls, the patient group showed significantly greater peak loading rates of the joint axial forces in the affected limb (*p* = 0.025–0.041) with a similar tendency in the unaffected limb during initial contact (Table 3).

For the vertical GRF at initial contact, in general, the peak loading and unloading rates were mainly correlated with the acetabular index of the unaffected side. Peak loading rates of both the affected and unaffected limbs were positively correlated with the acetabular index and c/b ratio of the unaffected side (Table 4). The peak unloading rates of both limbs were also positively correlated with the acetabular index of the unaffected side (Table 4). During loading response, the peak loading rates were about three times smaller than those at initial contact. The peak loading rates of the affected limbs were positively correlated with the acetabular index and c/b ratio of the unaffected side, but those during both initial contact and loading response phases were negatively correlated with the center-edge angle of the unaffected side (Table 4).

**TABLE 4:** Correlations between loading and unloading rates of vertical ground reaction force and radiographic measurements of both affected (A) and unaffected (U) sides.

		Peak loading rate (BW/s)		2nd peak loading rate (BW/s)		Peak unloading rate (BW/s)	
				
		Affected	Unaffected	Affected	Unaffected	Affected	Unaffected
Offset (mm)	*A*	–0.015	0.046	–0.018	0.086	0.252	0.364
	*U*	–0.191	–0.159	–0.154	–0.046	0.12	0.209
Neck-shaft angle (°)	*A*	0.122	–0.089	0.047	–0.187	–0.298	–0.345
	*U*	–0.407	–0.055	−**0.567**	0.116	–0.21	–0.315
Acetabular index (°)	*A*	0.135	0.256	0.095	0.263	0.393	0.353
	*U*	**0.801**	**0.632**	**0.740**	0.296	**0.548**	**0.595**
Center-edge angle (°)	*A*	–0.407	–0.126	–0.274	–0.185	–0.208	–0.217
	*U*	−**0.709**	–0.445	−**0.769**	–0.267	–0.308	–0.305
Acetabular depth ratio (mm/mm)	*A*	–0.141	–0.400	–0.299	−**0.490**	–0.149	–0.121
	*U*	–0.375	0.010	–0.437	–0.039	0.03	0.051
Articulotrochanteric distance (mm)	*A*	0.062	0.094	–0.046	0.072	0.02	–0.107
	*U*	–0.378	0.065	−**0.558**	0.134	0.004	–0.099
c/b ratio (mm/mm)	*A*	0.304	0.141	0.018	0.045	0.247	0.255
	*U*	**0.594**	**0.535**	**0.498**	0.432	0.436	0.418
Alsberg angle (°)	*A*	0.120	0.180	0.070	0.203	–0.202	–0.295
	*U*	–0.255	0.136	−**0.566**	0.156	–0.014	–0.088

*Bold: *p* < 0.05.*

*BW, body weight.*

For the joint axial forces during initial contact, the peak loading rates at the hip, knee and ankle on both sides were all positively correlated with the acetabular index of the unaffected side, while those on the affected side were correlated negatively with the center-edge angle of the unaffected side (Table 5). The peak axial force loading rate at the unaffected hip was also positively correlated with the Alsburg angle of the unaffected side, while those at the unaffected knee and ankle were positively correlated with the c/b ratio of the unaffected side. The axial force unloading rates at the knee and ankle on the affected side were positively correlated with the acetabular index of the unaffected side, while that at the ankle on the unaffected side was positively correlated with the acetabular index of the unaffected side. During loading response, the peak loading rates were about four times smaller than those at initial contact. The peak loading rates at the knee and ankle on the affected limb were negatively correlated with the acetabular depth ratio of the affected limb (Table 5).

**TABLE 5:** Correlations between loading and unloading rates of joint axial force and radiographic measurements of both affected (A) and unaffected (U) sides.

			Acetabular		Center-edge		Acetabular depth		c/b ratio		Alsberg	
			index (°)		angle (°)		ratio (mm/mm)		(mm/mm)		angle (°)	
							
			A	U	A	U	A	U	A	U	A	U
Hip	Loading, IC	*A*	0.003	**0.622**	–0.292	−**0.527**	–0.045	–0.214	0.307	0.359	–0.043	–0.216
		*U*	0.139	**0.541**	–0.096	–0.35	–0.423	–0.429	0.107	0.098	0.048	**0.491**
	Loading, LR	*A*	0.146	0.259	–0.216	–0.21	–0.471	–0.045	0.002	0.376	0.235	0.097
		*U*	0.398	0.274	–0.09	0.023	–0.392	0.065	0.108	0.121	–0.183	0.128
	Unloading	*A*	0.052	0.391	–0.004	–0.181	–0.089	–0.093	0.044	0.092	0.04	0.276
		*U*	0.094	0.362	–0.04	–0.199	–0.274	–0.072	0.033	0.301	–0.035	0.101
Knee	Loading, IC	*A*	–0.03	**0.613**	–0.262	−**0.552**	–0.043	–0.234	0.278	0.354	–0.001	–0.204
		*U*	0.092	**0.557**	–0.064	–0.372	–0.357	–0.181	0.024	**0.484**	0.264	0.251
	Loading, LR	*A*	0.095	0.275	–0.17	–0.246	−**0.526**	–0.095	–0.038	0.423	0.311	0.123
		*U*	0.406	0.331	–0.103	–0.059	–0.259	–0.027	0.184	0.161	–0.265	0.1
	Unloading	*A*	–0.021	**0.523**	–0.084	–0.383	–0.091	–0.093	0.073	–0.1	0.02	0.358
		*U*	0.002	0.457	–0.045	–0.341	–0.191	–0.202	0.019	0.322	–0.026	–0.012
Ankle	Loading, IC	*A*	0.008	**0.617**	–0.299	−**0.561**	–0.075	–0.234	0.305	0.369	0.016	–0.211
		*U*	0.122	**0.534**	–0.073	–0.334	–0.393	–0.138	0.025	**0.491**	0.268	0.284
	Loading, LR	*A*	0.14	0.258	–0.198	–0.201	−**0.559**	–0.104	–0.019	0.412	0.332	0.137
		*U*	0.326	0.303	–0.061	–0.094	–0.312	–0.181	0.318	0.243	–0.038	0.252
	Unloading	*A*	–0.164	**0.515**	–0.129	–0.527	–0.031	–0.037	0.09	–0.345	–0.05	0.323
		*U*	0.016	**0.502**	–0.12	–0.387	–0.249	–0.192	0.05	0.394	–0.016	–0.035

*IC, initial contact; LR, loading response phase.*

*Bold: *p* < 0.05.*

*Correlations coefficients of offset (mm), neck-shaft angle (°), and articulotrochanteric distance (mm): <0.5.*

## Discussion

The current study aimed to identify the loading and unloading rates of the GRF and joint axial forces in the lower limbs, as well as spatiotemporal parameters during gait, and the possible correlations of the loading and unloading rates with the bilateral hip morphology in juveniles who had been treated for DDH during toddlerhood. Compared to the healthy group, no significant between-group differences were found in most of the spatiotemporal parameters. The patients showed greater-than-normal peak loading rates of the joint axial forces on the affected side. Their vertical GRF and joint axial forces loading rates at initial contact on both the affected and unaffected sides were positively correlated to the acetabular index on the unaffected side. The peak unloading rates of the vertical GRF and the axial forces at the ankle and knee on the affected side, and at the ankle on the unaffected side were also positively correlated with the acetabular index on the unaffected side. These results suggest that apart from the regular follow-up of the morphological changes of the affected hip in current clinical practice, monitoring the loading rates and morphological changes of the unaffected hip after DDH reduction surgery is also important for early identification of any signs of insidious hip dysplasia and risk of premature degeneration of the cartilage.

Gait speed is a key factor affecting the magnitudes of the GRF (Cook et al., 1997) and the joint forces in the lower extremities (Paul, 1976; Röhrle et al., 1984), and their loading rates (Collins and Whittle, 1989) during level walking. However, gait speed and most spatiotemporal parameters did not appear to be an influencing factor for the observed between-group differences in loading and unloading rates in the current study as there were no significant between-group differences in gait speed. The only spatiotemporal parameter that was significantly different between groups was the stride time, but the differences were small and it was not directly related to the loading rates around heel-strike. These results suggest that early surgery for unilateral DDH was helpful for the overall gait efficiency as indicated by the patients’ normal gait speeds and spatiotemporal parameters (Table 2). It appears that the observed increases of the loading rates in the DDH group were more likely related to the control of the joints and end-point (foot) of the lower limbs around heel-strike and during the subsequent loading response as a result of the morphological conditions at the hips.

Compared to healthy controls, the juvenile subjects surgically treated during infancy for DDH displayed increased loading rates of the vertical GRFs and joint axial forces in the affected limb, but only showed a trend of increased loading rates in the unaffected limb during initial contact and loading response of gait. Similar findings were also found in adolescents who had been treated for DDH during infancy, but showing significantly increased loading rates in both the affected and unaffected limbs (Chang et al., 2011). In another study, osteonecrosis which developed in the treated hip of adolescents with DDH was found to lead to significantly greater loading rates during loading response in both affected and unaffected hips when compared with the adolescents with DDH but without osteonecrosis (Wang et al., 2016). Both the previous studies on adolescents suggested that the greater-than-normal loading rates in the affected and unaffected limbs were as a result of the compromised muscular control at the affected hip after surgery. However, this did not seem to explain the current findings in juveniles. The differences in the ages of gait experiment between Chang et al. (2011) and the current study may be a main factor for the differences in the findings. On the other hand, possible effects of the morphological changes of the hips were not considered in the previous studies. Nonetheless, from the previous and current results, it appears that risk of premature OA at the affected hip of the juvenile patients was already greater than for the healthy peers, but the risk at the unaffected hip was still less than that at the affected hip, and may be managed so as to avoid further deterioration over time.

In the current study, the loading rates of the vertical GRFs and joint axial forces at initial contact in both the affected and unaffected limbs were three to four times greater than those during loading response. Therefore, the correlations between the loading rates at initial contact and the hip morphology are more relevant to the risk of premature OA of the hip in the current patient population. At initial contact, the AI of the unaffected hip was the primary parameter that was positively correlated with the loading rates of the vertical GRFs and joint axial forces in both the affected and unaffected limbs. These loading rates also had weak to moderate, negative correlations with CEA of the unaffected hip. It is noted that even though good morphology was achieved after surgical treatment, a greater residual variability in the morphological features remained at the affected hip as indicated by the bigger standard deviations of the morphological parameters (Table 1). It is possible that these residual morphological variabilities at the affected hip reduced their correlations with the loading rates. While the loading rates and the AI of the unaffected hip were positively correlated, their causative relationships required further investigation. Nonetheless, such significant correlations may be explained from the point of view of the control of the swing-limb and the associated impulsive GRF at heel-strike.

Generally, the greater the AI, the more vertical the acetabulum and the worse the hip dysplasia (Albinana et al., 2004). Increased AI has also been shown to be related to shorter lever arms and thus impaired function of the abductors and flexors (Liu et al., 2012). Considering the kinetic chain from the stance limb to the swing foot, the unaffected hip is proximal to the swing knee and ankle, so increased AI of the unaffected hip will compromise the control of the rate at which the swing limb and the foot descend to the ground, leading to increased loading rates of the GRF. Since the time duration at IC (<10–20 ms) is much shorter than the time needed for the muscle to generate forces (23–73 ms) (Burke et al., 1971; Whittle, 1999), the muscle contractions are not fast enough to change their properties and the ability of attenuation in response to the impact. On the other hand, the longitudinal axes of the femur and tibia are aligned at heel-strike, which further reduces the ability of the muscles to attenuate the impact. Under these conditions, similar loading rates were transmitted upward from the ankle to the hip, which were all correlated to the AI of the unaffected hip (Tables 2, 3). Apart from loading rates, the acetabular index of the unaffected hip was also the primary parameter significantly correlated with the peak unloading rates of the vertical GRFs and axial forces at the knee and ankle in both limbs. This was expected because the leading limb accepted the body weight while the trailing limb released the body weight. Increases in both loading and unloading rates for both affected and unaffected limbs, and significant correlations between loading and unloading rates were also found in adolescent with surgically treated unilateral DDH (Chang et al., 2011; Wang et al., 2016).

The current results from juveniles and previous studies on adolescents suggest that there is an increased risk of further deterioration such as insidious dysplasia of both the affected and unaffected hips over time during growth in the juvenile patients. Therefore, measures should be taken to manage the condition of the unaffected hip and to prevent further increases in the loading rates in both the affected and unaffected limbs. Monitoring the loading and unloading rates in both limbs and morphological changes of both hips in juveniles after DDH reduction surgery is important for early identification of any signs of insidious hip dysplasia and risk of premature degeneration of the cartilage. In addition, the development of preventive strategies for the increased loading rates to reduce the risk of premature hip OA should be considered, such as the use of foot orthoses or footwear with shock-absorbing properties following surgery and rehabilitative gait retraining for developing gait patterns with reduced GRF loading rates.

The current study suggests that monitoring the morphological changes over time is equally important for both hips during post-operative follow-up in patients treated for DDH. Generally, morphological measurements are performed mainly on the affected hip in follow-up visits to identify reduction quality, residual deformity or any subsequent osteonecrotic changes, and include parameters such as acetabular index, neck shaft angle, articulotrochanteric distance (ATD), c/b ratio, and Alsberg angle. In a long-term follow-up of DDH after surgical reduction, 43% of OA change was on the affected side (Terjesen, 2018). However, the importance of follow-up of the morphology of the unaffected side was noted in only a limited number of studies, reporting insidious or occult hip dysplasia on the unaffected side (Jacobsen, 2006; Jacobsen et al., 2006; Song et al., 2008). In a 50-years follow-up study by Terjesen and Horn (2020) of 48 patients treated for unilateral DDH by, eight patients (17%) were reported to have developed dysplasia of the contralateral hip, six of whom had undergone surgery to improve the coverage of the femoral head. The results of these studies and the current findings of moderate to strong correlations between the loading rates in both hips and the acetabular index and CEA of the unaffected hip suggest that monitoring the morphological changes in the unaffected hip is important for attempting to prevent the development of gait patterns with increased loading rates on both the unaffected and affected hips as found in previous adolescent studies.

The current study was the first attempt to identify the correlational relationship between post-operative morphology of both hips and loading rates of the bilateral lower limb joints in juveniles treated for DDH during toddlerhood. For more definite causative conclusions, further longitudinal studies will be needed. Furthermore, since different surgical methods and the surgical age may have different effects on the post-operative condition and subsequent development of the morphology and mechanics of the hip (Wang et al., 2016; Scott et al., 2020) further affecting the loading conditions of the lower extremities, future studies on such effects will also be needed. On the other hand, the current study was limited to level walking. Further study on more challenging motor tasks such as stair-climbing and slope-walking (Liikavainio et al., 2007; Jeong and Shin, 2016) may help reveal other possible factors that may contribute to the development of gait patterns with increased loading rates at both hips in the current patient population.

## Conclusion

The juvenile patients treated for DDH during toddlerhood showed greater-than-normal peak loading rates of the joint axial forces, and the loading rates of the GRF and joint axial forces on both the affected and unaffected sides at initial contact were strongly correlated with the acetabular index on the unaffected side. The peak unloading rates on the affected side were also significantly correlated with the acetabular index on the unaffected side. These results suggest that apart from regular follow-up of the affected hip, routine assessment of the morphological changes of the unaffected hip and/or increased loading rates after DDH reduction surgery is important for early identification of any signs of insidious hip dysplasia and risk of premature degeneration of the cartilage.

## Data Availability Statement

The raw data supporting the conclusions of this article will be made available by the authors, without undue reservation.

## Ethics Statement

The studies involving human participants were reviewed and approved by Chang Gung Memorial Hospital Institutional Review Board (IRB No. 201601982B0C501). Written informed consent to participate in this study was provided by the participants’ legal guardian/next of kin.

## Author Contributions

W-CL, C-HC, T-MW, and T-WL conceived and designed the experiments. W-CL, T-YC, and L-WH performed the experiments and analyzed the data. W-CL, T-YC, and T-WL wrote the main manuscript text. T-WL, T-MW, and C-HC contributed subjects, materials, and analysis tools. All authors reviewed the manuscript.

## Conflict of Interest

The authors declare that the research was conducted in the absence of any commercial or financial relationships that could be construed as a potential conflict of interest.
